# Generalized min-max bound-based MRI pulse sequence design framework for wide-range *T*_1_ relaxometry: A case study on the tissue specific imaging sequence

**DOI:** 10.1371/journal.pone.0172573

**Published:** 2017-02-21

**Authors:** Yang Liu, John R. Buck, Vasiliki N. Ikonomidou

**Affiliations:** 1 Department of Electrical and Computer Engineering, University of Massachusetts Dartmouth, Dartmouth, MA, United States of America; 2 Department of Bioengineering, George Mason University, Fairfax, VA, United States of America; Henry Ford Health System, UNITED STATES

## Abstract

This paper proposes a new design strategy for optimizing MRI pulse sequences for *T*_1_ relaxometry. The design strategy optimizes the pulse sequence parameters to minimize the maximum variance of unbiased *T*_1_ estimates over a range of *T*_1_ values using the Cramér-Rao bound. In contrast to prior sequences optimized for a single nominal *T*_1_ value, the optimized sequence using our bound-based strategy achieves improved precision and accuracy for a broad range of *T*_1_ estimates within a clinically feasible scan time. The optimization combines the downhill simplex method with a simulated annealing process. To show the effectiveness of the proposed strategy, we optimize the tissue specific imaging (TSI) sequence. Preliminary Monte Carlo simulations demonstrate that the optimized TSI sequence yields improved precision and accuracy over the popular driven-equilibrium single-pulse observation of *T*_1_ (DESPOT1) approach for normal brain tissues (estimated *T*_1_ 700–2000 ms at 3.0T). The relative mean estimation error (MSE) for *T*_1_ estimation is less than 1.7% using the optimized TSI sequence, as opposed to less than 7.0% using DESPOT1 for normal brain tissues. The optimized TSI sequence achieves good stability by keeping the MSE under 7.0% over larger *T*_1_ values corresponding to different lesion tissues and the cerebrospinal fluid (up to 5000 ms). The *T*_1_ estimation accuracy using the new pulse sequence also shows improvement, which is more pronounced in low SNR scenarios.

## Introduction

Quantitative estimation of longitudinal relaxation time, termed *T*_1_ relaxometry, offers a very useful approach in magnetic resonance imaging (MRI) for enhancing tissue contrast, improving tissue characterization, and evaluating neuro-degenerative pathologies, etc. [1–3]. A *T*_1_ map shows more accurately the brain tissue characteristics when compared against more commonly used contrast-based qualitative approaches, such as *T*_1_/*T*_2_-weighted images. *T*_1_ relaxometry enables better tissue classification and holds promise for improving detection of early-stage tissue degeneration, as well as characterization of advanced tissue destruction [3]. Therefore, a fast, accurate, and precise *T*_1_ relaxometry technique can potentially be applicable in a variety of neuro-degenerative disorders, including multiple sclerosis (MS) [4], Alzheimer’s disease [5] and Parkinson’s disease [6], as well as in assisting image-guided surgeries.

*T*_1_ relaxometry techniques estimate *T*_1_ on a voxel-to-voxel basis using the magnetic signals acquired with specific MRI pulse sequences, which differ in their pulse times and flip angles. The pulse sequence parameters directly affect the acquired MR signals and thereby the performance of *T*_1_ relaxometry techniques. Estimating *T*_1_ by sampling the *T*_1_ relaxation signals has a five decade history [7–10]. The conventional methods are based on inversion recovery (IR) [7] and saturation recovery (SR) [8] due to their relatively large signal dynamic ranges. However, the clinical applications of IR and SR sequences are severely hampered by the considerable time required for the partial recovery of the longitudinal magnetization. To achieve a reasonable scan time, Look and Locker proposed a “one-shot” method, which samples multiple points along the *T*_1_ relaxation curve by continuously tipping the longitudinal magnetization with small flip angle pulses [9]. More recently, Deoni *et al*. presented a fast and high-resolution *T*_1_ mapping approach, coined driven-equilibrium single-pulse observation of *T*_1_ (DESPOT1) [10]. It uses a pair of spoiled gradient recalled echo (SPGR) images with different flip angles and achieves a shorter total scan time than other methods. However, researchers found that the *T*_1_ estimates using DESPOT1 are generally biased because the data processing does not properly account for noise [11]. This bias can be significant, such that *T*_1_ values are overestimated by as much as 10% to 20% for clinical SPGR images with *T*_1_ of 800–1600 ms [11]. Moreover, DESPOT1 is limited in its ability to estimate large *T*_1_ values corresponding to relatively advanced lesions and in its sensitivity to pulse flip angle perturbations [10].

Neuro-degenerative diseases like MS exhibit a wide range of damage to the brain’s white matter. Consequently, *T*_1_ relaxometry sequences must meet three requirements in order to be clinically useful. First, the technique needs to provide precise and accurate measurements in the range of *T*_1_s corresponding to normal white matter (WM, 800–900 ms at 3T) so as to identify early changes from the ‘normal’ condition. Second, the technique needs to be stable for measuring values up to those corresponding to cerebrospinal fluid (CSF, 4500–5500 ms at 3T) in order to be able to characterize the degree of damage in more advanced lesions. Third, the technique needs to do so within clinically acceptable scan times (of the order of 10–15 minutes) while being reasonably stable to variations of the radiofrequency (*B*_1_) field. Currently, no existing technique fulfills all three requirements. These technological limitations preclude the use of *T*_1_ relaxometry as a tool that may address both early white matter degeneration as well as lesion evolution, which are both crucial markers for monitoring the performance of neuroprotective drugs in treating dementia and progressive MS.

This paper proposes a new pulse sequence design framework for optimizing the *T*_1_ relaxometry performance for a broad range of *T*_1_ values. There are two major components of the proposed framework that differ from previous ones. The first is to use the Cramér-Rao bound (CRB) to design the pulse sequence. The CRB provides a lower bound on the variance of any unbiased *T*_1_ estimate. This bound-based design allows us to predict the performance of an MRI sequence based on its sequence parameters, and then to optimize the performance of that sequence by adjusting these parameters. Our CRB derivation leads to a geometric interpretation, which brings into sharper focus all of the factors that control the precision of *T*_1_ relaxometry sequences. This differs from previous sequence design approaches that merely focus on increasing the dynamic range and signal-to-noise ratio (SNR) to improve *T*_1_ mapping performance [10, 12, 13]. The second novel component of our approach is to design the pulse sequence by optimizing its performance over a broad range of *T*_1_ values. Previous sequence design algorithms optimized the signal dynamic range or sensitivity for a single nominal *T*_1_ value, or a small range of *T*_1_ values [10–13]. When the tissue relaxation times fell outside the narrow range of nominal values, the relaxometry performance degraded rapidly. In contrast, our framework employs a min-max optimization strategy, which designs the pulse sequence to minimize the maximum estimate variance over a broad range of *T*_1_ values. This guarantees the overall optimality of the resulting sequence for all *T*_1_ values within the target region spanning thousands of milliseconds.

To demonstrate the effectiveness of the proposed sequence design framework, we optimize the tissue specific imaging (TSI) sequence for *T*_1_ relaxometry. The proposed technique is sufficiently general that it can optimize nearly any *T*_1_ relaxometry sequence. Optimizing the TSI sequence provides a powerful example of the benefits of bound-based min-max optimization because it yields three high-contrast images of WM, gray matter (GM) and CSF, which are employable as anatomical references in clinical settings. The TSI sequence is a relatively new imaging sequence and has been successfully applied for characterization of MS lesions [14, 15]. The TSI pulse sequence includes three imaging pulses followed EPI acquisitions, which are interleaved with two inversion pulses in each pulse repetition period. This sequence was originally designed to acquire brain images for each of the two categories of brain tissues (WM and GM), as well as the CSF, with optimal contrast. We address the potential applications of TSI for *T*_1_ relaxometry and optimize its sequence parameters to improve the *T*_1_ estimate precision and accuracy while maintaining its total scan time. Another motivation for applying the TSI-type sequences for *T*_1_ relaxometry is their improved precision relative to DESPOT1 over the *T*_1_ range corresponding to normal brain tissues, and, more importantly, their improved stability for larger *T*_1_ values corresponding to tissues such as advanced MS lesions [16, 17].

## Theory

### MR signal model

*T*_1_ relaxometry approaches apply the RF pulse sequences repeatedly to initialize the magnetization preparation. Denote the MR signal generated by a tissue voxel at time *t*_*i*_ as *x*_*i*_ = *M*_0_h_*i*_(*T*_1_), *i* = 1, 2, …, *n*, where *M*_0_ is the equilibrium longitudinal magnetization and *h*_*i*_(*T*_1_) is the signal weighting factor at time *t*_*i*_. The acquired signal at time *t*_*i*_ follows

$$s_{i}=M_{0}{\text{h}}_{i}\left(T_{1}\right)+w_{i},$$ (1)

with additive uncorrelated noise *w*_*i*_ for each acquisition. The vector **s** = [*s*_1_, …, *s*_*n*_]^*T*^ characterizes the acquired signals at different times *t*_1_, …, *t*_*n*_, where (⋅)^*T*^ denotes vector transpose. Eq (1) is known as a universal MR signal acquisition model assuming additive noise [25]. Assuming knowledge of the pulse sequence parameters (sequence repetition time TR, pulse times *t*_*i*_ and flip angles *α*_*i*_), the signal weighting factor as a function of *T*_1_ after the *n*th pulse can be derived from the Bloch equations in Eq (2), where $M_{z}^{eq}$ is the steady state magnetization. To derive $M_{z}^{eq}$, we assume that in each TR, the first imaging pulse is applied at the initial time *t* = 0 and there is a delay of (TR − *t*_*n*_) after the *n*th pulse of one sequence and before the first pulse of the next sequence.

$${\text{h}}_{n}\left(T_{1}\right)=\sin\alpha_{n}\left\{1+\left[\sum_{i=1}^{n-2}\left(\cos\alpha_{i}-1\right)\left(\prod_{j=i+1}^{n-1}\cos\alpha_{j}\right)e^{\frac{t_{i}}{T_{1}}}+\left(\cos\alpha_{n-1}-1\right)e^{\frac{t_{n-1}}{T_{1}}}+\left(\frac{M_{z}^{eq}}{M_{0}}-1\right)\prod_{i=1}^{n-1}\cos\alpha_{i}\right]e^{-\frac{t_{n}}{T_{1}}}\right\}.$$ (2)

$$M_{z}^{eq}=M_{z}^{0}\frac{\left(1+e^{-\text{TR}/T_{1}}\left\{\sum_{i=1}^{N-1}\left(\cos\alpha_{i}-1\right)\left(\prod_{j=i+1}^{N}\cos\alpha_{j}\right)e^{t_{i}/T_{1}}+\left(\cos\alpha_{N}-1\right)e^{t_{N}/T_{1}}-\prod_{i=1}^{N}\cos\alpha_{i}\right\}\right)}{\left(1-e^{-\text{TR}/T_{1}}\prod_{i=1}^{N}\cos\alpha_{i}\right)}.$$ (3)

The MR signals are most commonly acquired through quadrature detector channels, each of which typically suffer from independent additive zero mean, white and Gaussian noise. Thus, the noise in the reconstructed magnitude MR images should follow a Rician distribution [18]. Several references in the MRI literature use Rician distributed random noise in their signal models [19, 20]. When the signal-to-noise ratio (SNR) is high enough, the Rician distribution converges to the Gaussian distribution [21, 22]. For the convenience of the CRB evaluation, we assume the SNR is sufficiently high to exploit the approximation of the noise as additive zero mean, white and Gaussian noise in the signal model in Eq (1). As will be shown later, this Gaussian model assumption leads to a readily-derived closed-form expression to geometrically interpret the CRB, which provides insight into the factors controlling the *T*_1_ estimate precision.

### Cramér-Rao bound on joint *M*_0_ and *T*_1_ estimation

The CRB has been used as a quantitative tool for optimizing experimental MR protocols [20, 23, 24] and also evaluating the precision of specific relaxometry sequences [25–27]. The analytic expression for the CRB on the *T*_1_ estimate, when jointly estimating *M*_0_ and *T*_1_, is the foundation for the sequence design framework. Letting the parameter vector *θ* = [*M*_0_, *T*_1_]^*T*^, the covariance matrix $C(\hat{\theta })$ of the unbiased estimator $\hat{\theta }$ satisfies

$$C(\hat{\theta })-I^{-1}\left(\theta \right)\geq 0,$$ (4)

where **I**(*θ*) is the 2 × 2 Fisher information matrix (FIM) [28]. As a consequence, the variance of any unbiased estimation of *T*_1_ is bounded from below by the (2,2) entry of matrix **I^−1^**(*θ*). This quantity is also known as the CRB of the *T*_1_ estimate

$$\text{Var}\left({\hat{T}}_{1}\right)\geq {\left(I^{-1}\left(\theta \right)\right)}_{22}=\text{CRB}\left(T_{1}\right).$$ (5)

The diagonal elements of the FIM represent the measured signals’ sensitivity to the parameters in *θ* [28]. For joint estimation of *M*_0_ and *T*_1_, the derivation of the FIM is straightforward following Eqs (6) and (7)

$$I\left(\theta \right)=E\left(\left[\nabla_{\theta }\ln p\left(s;\theta \right)\right]{\left[\nabla_{\theta }\ln p\left(s;\theta \right)\right]}^{T}\right),$$ (6)

where *E*(⋅) takes the expectation over the acquired signal **s**. Eq (6) has entries

$$\begin{array}{lll} I_{11} & = & \frac{1}{\sigma^{2}}\sum_{i=1}^{n}{\left({\text{h}}_{i}\left(T_{1}\right)\right)}^{2},\\ I_{12}=I_{21} & = & \frac{1}{\sigma^{2}}\sum_{i=1}^{n}\left({\text{h}}_{i}\left(T_{1}\right)\frac{\partial {\text{h}}_{i}\left(T_{1}\right)}{\partial T_{1}}M_{0}\right),\\ I_{22} & = & \frac{1}{\sigma^{2}}\sum_{i=1}^{n}{\left(M_{0}\frac{\partial {\text{h}}_{i}\left(T_{1}\right)}{\partial T_{1}}\right)}^{2}, \end{array}$$ (7)

where *σ* is the standard deviation of the additive noise *w*. Note that the dependence of the FIM on the pulse sequence parameters TR, *t*_1_, …, *t*_*N*_ and *α*_1_, …, *α*_*N*_ is suppressed here for brevity. Defining SNR = *M*_0_/*σ* yields the closed-form expression for the CRB on *T*_1_ estimate

$$\text{CRB}\left(T_{1}\right)=\frac{{\left(\text{SNR}\right)}^{-2}}{\left(\sum_{i=1}^{n}{\left(\frac{\partial {\text{h}}_{i}\left(T_{1}\right)}{\partial T_{1}}\right)}^{2}-\frac{{\left(\sum_{i=1}^{n}{\text{h}}_{i}\left(T_{1}\right)\frac{\partial {\text{h}}_{i}\left(T_{1}\right)}{\partial T_{1}}\right)}^{2}}{\sum_{i=1}^{n}{\left({\text{h}}_{i}\left(T_{1}\right)\right)}^{2}}\right)}.$$ (8)

**Fig 1:**
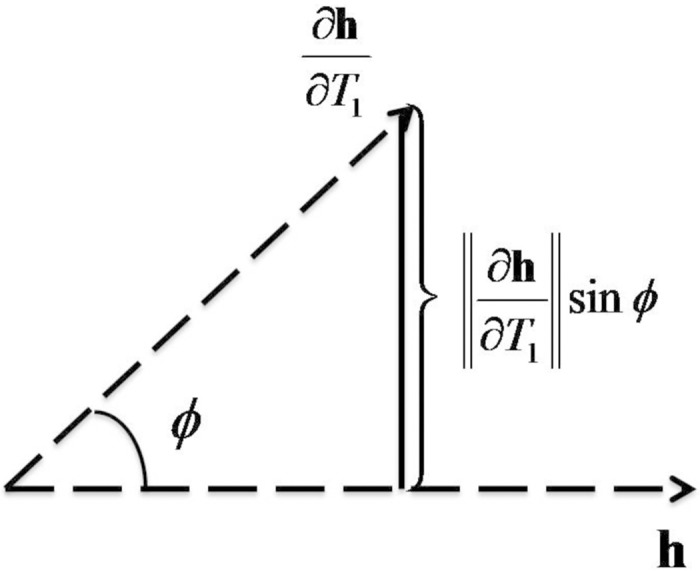
Geometric interpretation of the Cramér-Rao Bound (CRB) of *T*_1_ estimate in a linear space. **h** is the signal weighting vector containing the measured signals at all acquisition times. *∂***h**/*∂T*_1_ is the sensitivity vector, calculated as the derivative of the signal weighting vector with respect to *T*_1_. Conceptually, increasing the norm of the sensitivity term ∥*∂***h**/*∂T*_1_∥ will increase the impact of small changes in *T*_1_ on the acquired signals. The orthogonality term sin *ϕ* is a consequence of the joint estimation of *T*_1_ and *M*_0_. The observed signal’s sensitivity for *M*_0_ is **h**, while that of *T*_1_ is *∂***h**/*∂T*_1_. The more orthogonal these vectors are, the easier it becomes to ascribe changes in the observed signal to *M*_0_ or *T*_1_ unambiguously.

To maximize the precision of the *T*_1_ estimate, we should choose the sequence parameters to minimize Eq (8). The apparent complexity of Eq (8) can be simplified through a linear space interpretation [29]. To achieve this, define the signal weighting vector as **h**(*T*_1_) = [h_1_(*T*_1_), h_2_(*T*_1_), …, h_*n*_(*T*_1_)]^*T*^, and the sensitivity vector ∂**h**/∂*T*_1_ as the derivative of **h** with respect to *T*_1_. Moreover, define *ϕ* as the principal angle between the vectors **h** and ∂**h**/∂*T*_1_ in the linear space (see Fig 1). Note that the principal angle *ϕ* between two vectors **x** and **y** in the linear space is defined as $\phi =\arccos\frac{\langle x,y\rangle }{∥x∥\cdot ∥y∥}$, where 〈⋅〉 is the inner product operator and ‖⋅‖ is the Euclidean norm (or length) of a vector. Therefore, Eq (8) can be rewritten as

$$\begin{array}{ccc} \text{CRB}(T_{1}) & = & {\left(\text{SNR}\right)}^{-2}{\left({\left|\left|\frac{\partial h}{\partial T_{1}}\right|\right|}^{2}-\frac{{\left(h^{T}\frac{\partial h}{\partial T_{1}}\right)}^{2}}{∥h{∥}^{2}}\right)}^{-1}\\ & = & {\left(\text{SNR}\left|\left|\frac{\partial h}{\partial T_{1}}\right|\right|\sin\phi \right)}^{-2}. \end{array}$$ (9)

Eq (9) makes clear that three factors control the CRB and thus there are three methods to improve the *T*_1_ estimate precision. The first method is to increase the SNR, which is consistent with previous sequence designs seeking to increase the signal dynamic range or reduce the noise [10, 12]. These approaches would improve *T*_1_ estimate stability, but are not always the most effective methods of reducing variance. The second term ∂**h**/∂*T*_1_ in Eq (9) is the sensitivity of *T*_1_, which describes how sensitive the signal model is to *T*_1_ variation. Increasing the norm of the sensitivity ∥∂**h**/∂*T*_1_∥ will increase the impact of small changes in *T*_1_ on the overall signal weighting vector **h**. For example, a large sensitivity indicates that a small *T*_1_ variation causes a large signal fluctuation. In this case, *T*_1_ can be estimated from the signal measurements more precisely. In contrast, zero sensitivity indicates that the signal will remain constant regardless of the *T*_1_ variation. In this case, the *T*_1_ value can never be estimated from the measurements. Increasing the magnitude of the sensitivity vector can be an effective method of improving *T*_1_ estimate precision. The third component in the CRB expression is the orthogonality term sin *ϕ*, which results from jointly estimating *T*_1_ and *M*_0_. The observed signals’ sensitivity for *M*_0_ is **h**, while that of *T*_1_ is ∂**h**/∂*T*_1_. The more orthogonal these vectors are, the easier it becomes to ascribe changes in the observed signal to *M*_0_ or *T*_1_ unambiguously. In terms of *T*_1_ estimation, increasing the orthogonality of the signal weighting vector and the sensitivity vector improves the *T*_1_ estimate precision. The second and third approaches are notable as they can be exploited without requiring the costly hardware improvements usually needed to increase signal strength or decrease measurement noise. To the best of our knowledge, no prior MRI sequence design strategy has exploited these mechanisms simultaneously and analyzed these approaches explicitly for improving the precision of *T*_1_ relaxometry.

## Methods

### Sequence optimization methods

As Eq (8) shows, the CRB is a function of the true *T*_1_ value. Current publications suggest that the variation of *T*_1_ within normal and diseased tissues scales with the mean of *T*_1_. For example, clinical *T*_1_ measurements at 1.5T using histograms of normal-appearing white matter (NAWM) showed *T*_1_ of 792 ms ± 36 for patients with secondary progressive MS. This represents a relative *T*_1_ variation of ± 4.5% around its mean. However, histograms for the cortical normal-appearing gray matter (NAGM) showed *T*_1_ of 1355 ms ± 62 for patients with secondary progressive MS, which also represents a relative *T*_1_ variation of ± 4.5% around its mean [30, 31]. In this example, the same stage of MS development corresponds to the same relative *T*_1_ error for different tissue types. Therefore, rather than directly using the CRB, this paper uses a relative error as the metric for optimizing the pulse sequence parameters. The relative error is defined as the square root of the CRB normalized by the true *T*_1_ value. The sequence parameters are optimized to minimize the maximum relative error over a broad range of *T*_1_ values. This guarantees the relative error to be reasonably robust to the *T*_1_ range of interest. For a given set of pulse times **t** = [*t*_1_, *t*_2_, …, *t*_*i*_], sequence repetition time TR, pulse flip angles ***α*** = [*α*_1_, *α*_2_, …, *α*_*i*_], and unknown parameters ***θ*** = [*M*_0_, *T*_1_]^*T*^, the maximum relative error over the range of ***θ*** follows

$${\text{C}}_{\text{max}}\left(t,\text{TR},\alpha \right)=\max_{\theta_{\text{lower}}\leq \theta \leq \theta_{\text{upper}}}\sqrt{\text{CRB}(t,\text{TR},\alpha ,\theta )}/T_{1}.$$ (10)

**Fig 2:**
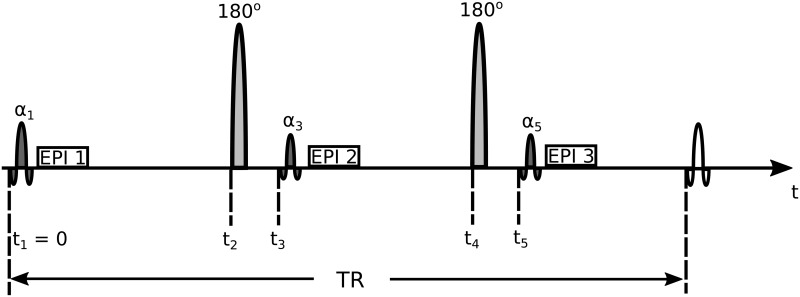
The general pulse sequence scheme for tissue specific imaging (TSI). In each TR period, there are three imaging pulses (dark gray) followed by EPI acquisitions and interleaved by two inversion pulses (light gray) (After Fig 1 from [14]). The three imaging pulses are characterized by their flip angles *α*_1_, *α*_3_, *α*_5_. The dashed lines indicate the times *t* when each pulse is applied. The first imaging pulse is applied at the beginning of each TR (*t*_1_ = 0). There are 8 pulse parameters to optimize for in the TSI sequence: times for the two inversion pulses *t*_2_, *t*_4_, times for the second and third imaging pulses *t*_3_, *t*_5_, flip angles of the three imaging pulses *α*_1_, *α*_3_, *α*_5_ and the sequence repetition time TR.

Fig 2 illustrates the structure of the TSI pulse sequence [14]. In each TR, there are three imaging pulses each followed by EPI acquisitions and interleaved by two inversion pulses. The sequence parameters were derived from a simulated annealing optimization process by maximizing the contrast-to-noise ratio (CNR) in the combined tissue-specific images. Using 3D sensitivity encoded (SENSE) EPI for data acquisition, the sequence achieves a 1.15 mm isotropic resolution in a FOV of 220 × 165 × 110 mm^3^ within a scan time of 10 minutes. There are eight pulse parameters to optimize in this sequence: times for the two inversion pulses *t*_2_, *t*_4_, times for the second and third imaging pulse *t*_3_, *t*_5_, flip angles for the three imaging pulses *α*_1_, *α*_3_, *α*_5_ and the sequence repetition time TR. The optimization process assumes the *T*_1_ interval of interest to be 700–2000 ms for normal brain tissues at 3.0T [32]. However, the performance of the optimized pulse sequence is evaluated over a broader *T*_1_ range of 700–5000 ms. This is to observe the sequence’s robustness to larger *T*_1_ variations that characterize more advanced lesions and the CSF region [3]. To achieve optimal sequence parameters that are practically applicable, we constrain the optimization process following the discussions in [14]. Specifically, the maximum allowed TR is set as 6 seconds to limit the total scan time to under 10 min. The inter-pulse interval needs at least 100 ms to allow enough time for the EPI acquisition. The flip angles of all imaging pulses are less than 90° for the convenience of practical implementation. More explicitly, the optimal pulse parameters are achieved by minimizing C_max_(**t**, ***α***) under the following constraints

$$\begin{array}{ccc} {\left(t,\alpha \right)}^{\text{opt}} & = & \arg\min_{t,\alpha }\;\;{\text{C}}_{\text{max}}\left(t,\alpha \right);\\ s.t.\;\;\;\text{TR} & \leq & 6\;\text{s},\\ t_{i}-t_{i-1} & \geq & 100\;\text{ms},\\ \text{TR}-t_{5} & \geq & 100\;\text{ms},\\ \alpha_{1},\alpha_{3},\alpha_{5} & \in & [0^{{}^{\circ}},90^{{}^{\circ}}],\\ \alpha_{2} & = & \alpha_{4}=180^{{}^{\circ}}. \end{array}$$ (11)

Due to the large number of parameters to optimize, classical optimization methods such as exhaustive search can be too computationally burdensome and gradient search can fail to converge to the globally optimal solution. To find the globally optimal sequence parameters, we adopt a hybrid of the Nelder-Mead downhill simplex method [33] and the simulated annealing approach [34]. The downhill simplex method finds the local minimum by expanding, contracting, and reflecting the simplex constructed by a group of different initial points in the N-dimensional hyperspace. With simulated annealing, the algorithm conditionally accepts uphill movement (leading to worse performance) during the optimization process and therefore improves the probability of finding the global optimum. This hybrid approach [35] has been proven successful in optimizing brain tissue contrast [14] and acquisition schemes for quantitative magnetization transfer MRI [36].

The optimization process is initialized with a TR of 5500 ms, evenly spread-out pulse times within TR, and a flip angle of 45° for each imaging pulse. For each temperature *T*, the simplex routine iterates 2000 times to calculate the cost function in Eq (10). For each iteration, the relative error is calculated over the *T*_1_ range 700–2000 ms, where the worst cases of the relative errors are compared and arranged in an ascending order for all simplex vertices. The relative error is randomly perturbed by a quantity *T* ⋅ log(*p*), where *p* is a random variable uniformly distributed within [0, 1], to allow for conditional acceptance of uphill movement [34]. The temperature *T* and the simplex size *D* decrease according to the annealing schedule

$$\begin{array}{lll} T\left(n+1\right) & = & 0.985T\left(n\right)\\ D\left(n+1\right) & = & 0.998D\left(n\right). \end{array}$$ (12)

The initial temperature is selected empirically as 0.8 and the initial simplex is constructed with a simplex size of 100 ms for the pulse times and 30° for the flip angles. The overall simulated annealing algorithm runs for 1000 iterations to locate a hopefully global optimum. During the optimization process, a prohibitive penalty of 10^3^ is added to the cost function whenever the new pulse sequence violates the constraints in Eq (11). Repeating the optimization process from several different initial sequence vectors allowed the pulse sequence to converge to a relatively stable and hopefully globally optimal CRB.

### *T*_1_ estimation methods

In general, there are two criteria to evaluate the performance of an estimator given an acquired signal **s**: accuracy and precision [28]. The accuracy of a *T*_1_ estimator ${\hat{T}}_{1}=f\left(s\right)$ is measured by the bias

$$\text{Bias}\left({\hat{T}}_{1}\right)=E\left({\hat{T}}_{1}\right)-T_{1},$$ (13)

which is the difference between the mean of the *T*_1_ estimator $E({\hat{T}}_{1})$ and the true *T*_1_. The smaller the bias is, the more accurate the estimator ${\hat{T}}_{1}$ is. The precision is determined by the variance of ${\hat{T}}_{1}$

$$\text{Var}\left({\hat{T}}_{1}\right)=E\left[{\left({\hat{T}}_{1}-E\left({\hat{T}}_{1}\right)\right)}^{2}\right]$$ (14)

The smaller the variance is, the more precise the estimator ${\hat{T}}_{1}$ is. An ideal *T*_1_ estimator ${\hat{T}}_{1}$ is unbiased, with its variance achieving the CRB of *T*_1_. For the signal model in Eq (1), the least squared estimator (LSE) of *T*_1_ for joint *M*_0_ and *T*_1_ estimation is asymptotically optimal. For infinitely high SNR, this estimator has its expected value achieve the true *T*_1_ and its variance achieve the CRB(*T*_1_) [28]. Mathematically, the LSE of ***θ*** = [*M*_0_, *T*_1_] minimizes the squared error

$$J\left(\theta \right)={\left(s-x\left(\theta \right)\right)}^{T}\left(s-x\left(\theta \right)\right),$$ (15)

where **x**(***θ***) describes the noise-free signal. Specifically, when the signal model shows linearity in one parameter *M*_0_ and non-linearity in *T*_1_, the LSE of *T*_1_ can be calculated in a more computationally efficient way by minimizing another version of squared error [28] following

$$J\left(T_{1}\right)=s^{T}\left[I-h{\left(h^{T}h\right)}^{-1}h^{T}\right]s,$$ (16)

where **I** is the identity matrix and **h** is the signal weighting vector defined in Eq (2). Since the squared error in Eq (16) depends only on one parameter *T*_1_, it is easy to find the *T*_1_ value which minimizes the squared error using a grid search of *T*_1_ in an appropriate range.

### Numerical simulation methods

The accuracy and precision of *T*_1_ estimates are evaluated in Monte Carlo simulations. The simulations consider four different *T*_1_ estimation approaches: nonlinear least square estimation (NLSE) using the optimized TSI sequence, NLSE using the original TSI sequence [14], linear LSE using the SPGR sequence (also coined DESPOT1 [10]), and NLSE using the SPGR sequence [11]. The observed magnetic signals were simulated for an equilibrium longitudinal magnetization value of *M*_0_ = 3000 a.u and a range of *T*_1_ within 700–5000 ms covering almost all brain tissues and CSF at 3.0T [32]. The different structures of the TSI and SPGR sequences require different approaches to simulate the observed noise-free signals. For the TSI signals, the steady state magnetizations in Eq (3) are first evaluated based on different choices of TSI sequence parameters. The signal weighting factor in Eq (2) is then evaluated at different pulse times and scaled by *M*_0_ as the raw noise-free magnetic signals. In contrast, the raw SPGR signals are simulated by evaluating

$$S_{\text{SPGR}}=\frac{M_{0}\left(1-E_{1}\right)\sin\alpha }{1-E_{1}\cos\alpha }$$ (17)

with $E_{1}=e^{-\text{TR}/T_{1}}$ at a given constant TR and varying flip angles [10]. For both TSI and SPGR, the simulated magnetic signals are then generated by adding varying levels of white Gaussian noise.

To accurately simulate and compare the performance of TSI and SPGR sequences on the same MRI machine with the same magnet and sensing coils, the SNR levels must be adjusted between the two sequences to account for the differences in their physical scan parameters. The simulated SNR is calculated as the ratio between *M*_0_ and the noise standard deviation *σ*. To enable a fair comparison, we assume the *B*_0_ strength, the voxel size, and the number of data measurements to be the same between TSI and SPGR. This assumption leaves out three factors for calibration: receiver bandwidth BW, $T_{2}^{*}$ relaxation time decay, and sensitivity encoded (SENSE) EPI for TSI. A larger BW incorporates more noise in the acquired signals and therefore decreases the SNR by a relative factor of $\sqrt{\text{BW}}$ [37]. A longer echo time *T*_*E*_ in gradient echo imaging decreases the acquired signal amplitude and therefore decreases the SNR through $e^{-T_{E}/T_{2}^{*}}$. For SPGR, the chosen pulse parameters are TR = 7.8 ms, *T*_*E*_ = 2.4 ms and receiver BW of ±31.3KHZ [38]. For TSI, the chosen pulse parameters are TR = 6 s, *T*_*E*_ = 35 ms and readout time of 2.048 *μ*s/sample (equivalent to receiver BW of ±244.1KHZ) [14]. The BW of TSI is eight times larger than that of SPGR, resulting in a SNR for TSI of $\sqrt{8}$ lower than SPGR. Assuming an average $T_{2}^{*}$ value of 48.9 ms for brain parenchyma [39], the relative $T_{2}^{*}$ decay gives TSI a lower SNR than SPGR by a factor of $e^{-\left({\text{TE}}_{\text{TSI}}-{\text{TE}}_{\text{SPGR}}\right)/T_{2}^{*}}=e^{-(35-2.4)/48.9}\;\approx \;0.5$. The SENSE EPI rate of 2 will further decrease the SNR of TSI by a factor of $\sqrt{2}$ due to fewer phase encoding (PE) steps during signal acquisitions [37]. Combining all these factors, the simulations of SPGR signals must have a SNR level 8 times greater than TSI to match the simulated performances with practical experiments.

## Results

### Pulse sequence optimization results

The main result of applying the proposed sequence design framework is that the optimized TSI pulse parameters achieve improved precision and accuracy over both the original TSI sequence [14] and the DESPOT1 sequence [10]. Table 1 shows the optimized TSI sequence parameters (TSI_new_) obtained by the optimization algorithm in the Methods section, compared against the original TSI sequence from [14]. The original TSI sequence was obtained assuming nominal tissue *T*_1_ values for WM of 800 ms, GM of 1550 ms and CSF of 3700 ms. In contrast, the optimization process assumes a range of *T*_1_ values 700–2000 ms for the normal brain tissues. Table 1 shows that the parameters of TSI_new_ differ from the TSI_original_ in the pulse times and flip angles. For TSI_new_, all pulses occur in the first 3655 ms over a TR of 6 seconds, leaving a relatively longer time for the longitudinal magnetization to relax after the third imaging pulse. In contrast, the pulses in the original TSI sequence are more spread out over TR. Moreover, the flip angles of TSI_new_ increase across the sequence, while the flip angles of TSI_original_ first decrease then increase.

**Table 1:** Optimized TSI pulse sequence parameters (TSI_new_), compared against the original TSI pulse parameters from Table 2 of [14].

	Imaging pulse 1	Inversion pulse 1	Imaging pulse 2	Inversion pulse 2	Imaging pulse 3
TSI_new_	0 ms/ 24°	1804 ms	2751 ms/ 68°	3555 ms	3655 ms/ 87°
TSI_original_	0 ms/ 46°	3020 ms	3573 ms/ 23°	5112 ms	5575 ms/ 83°

### *T*_1_ estimation performances: Precision and accuracy

To evaluate and compare the *T*_1_ estimate precision and accuracy, we designed two Monte Carlo simulation experiments involving four estimators: NLSE with the new TSI sequence, NLSE with the original TSI sequence, DESPOT1, and NLSE with the SPGR sequence used by DESPOT1. The precision is compared in terms of the relative mean estimation error: the standard deviation of the estimated *T*_1_ over the true *T*_1_. Five thousand Monte Carlo trials are repeated for each set of pulse parameters for each value of *T*_1_. Equivalent SNR levels are calibrated as SNR = 125 for the TSI sequences and SNR = 1000 for the SPGR sequence. For both TSI methods and SPGR methods, the relative mean estimation errors are compared against their own theoretical lower bounds, or relative errors, calculated as the square root of the CRB over the true *T*_1_.

**Fig 3:**
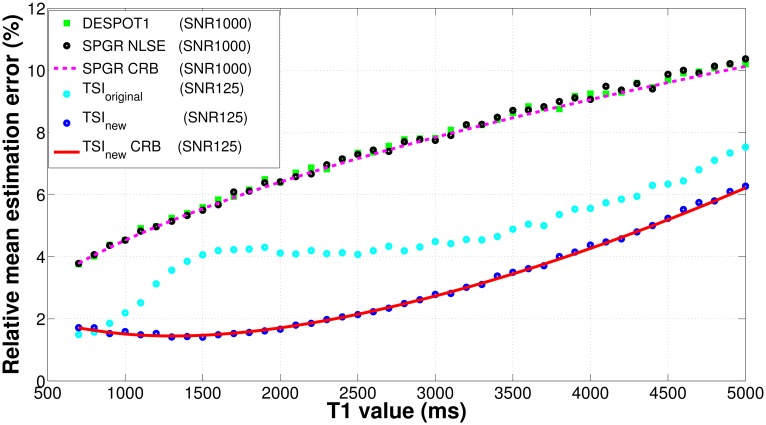
Comparing *T*_1_ estimates’ precision for four different approaches: NLSE with the new TSI sequence (blue) against its theoretical lower bound (red solid), NLSE with the original TSI sequence (cyan), DESPOT1 (green), and NLSE with the SPGR sequence (black) against its theoretical lower bound (magenta dashed). The precision is measured in terms of the relative mean estimation error, calculated as the standard deviation of *T*_1_ estimates normalized by the true *T*_1_. The theoretical lower bound of the relative mean estimation error is the relative error, calculated as the square root of the CRB on *T*_1_ estimates normalized by the true *T*_1_. SNR levels equalizing for both receiver bandwidths and echo times are calibrated as SNR = 125 for the TSI sequences and SNR = 1000 for the SPGR sequence. The new TSI sequence achieves the lowest mean estimation error and therefore highest precision for tested *T*_1_ values.

Fig 3 compares the *T*_1_ estimate precision in terms of the relative mean estimation errors for the four different estimators. For the evaluated *T*_1_ range of 700–5000 ms, the TSI sequences show overall improvement over the SPGR sequence. The new TSI sequence achieves mean estimation error less than 1.7% for normal brain tissues (*T*_1_ 700–2000 ms). The new TSI sequence also maintains the best robustness to *T*_1_ variation, with mean estimation error less than 6.5% when *T*_1_ reaches 5000 ms. In the SPGR family, DESPOT1 and SPGR NLSE provide similar errors: less than 7.0% for *T*_1_ of 700–2000 ms (agreeing with the findings in [10, 11]) and less than 10.5% when *T*_1_ reaches 5000 ms. Both SPGR NLSE and TSI_new_ NLSE achieve their theoretical lower bounds for all tested *T*_1_ values. This implies that the CRB provides a reliable prediction of the precision performance for different pulse sequences.

A second Monte Carlo experiment compares the accuracy of *T*_1_ estimates among the four approaches over a broad range of SNRs. The accuracy is measured in terms of the $\text{relative bias}:\;|(\text{Mean of }{\hat{T}}_{1}\;-\;{\text{True }T}_{1}{)/\text{True }T}_{1}|$. This experiment uses a nominal *T*_1_ value of 1500 ms, which is a typical *T*_1_ value for brain GM at 3.0T. Five thousand Monte Carlo trials are repeated for each set of pulse parameters for each SNR level. Again, to calibrate for the SNR equivalence between the TSI sequences and the SPGR sequence, simulated SNRs are selected with 5 ≤ SNR ≤ 60 for TSI sequences and 40 ≤ SNR ≤ 480 for both DESPOT1 and SPGR NLSE.

**Fig 4:**
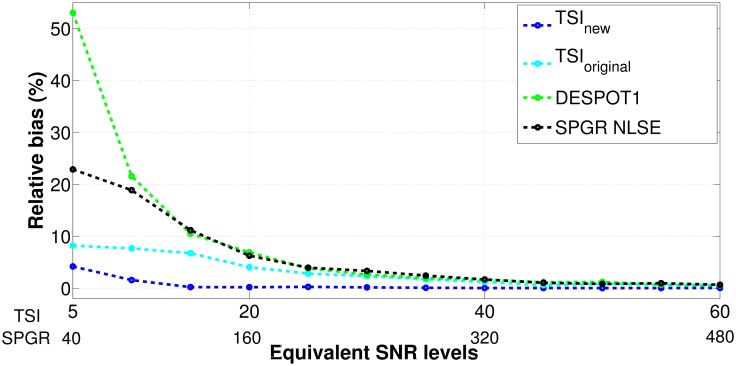
Comparing the *T*_1_ estimates’ accuracy for four different approaches: NLSE with the new TSI sequence (blue), NLSE with the original TSI sequence (cyan), DESPOT1 (green), and NLSE with the SPGR sequence (black). The accuracy is measured in terms of the relative bias %, calculated as $\left|(\text{Mean of }{\hat{T}}_{1}\;-\text{ True }T_{1})/\text{True }T_{1}\right|$. This experiment uses a nominal *T*_1_ value of 1500 ms. Simulated SNR levels are calibrated equivalently as 5 ≤ SNR ≤ 60 for the TSI sequences and 40 ≤ SNR ≤ 480 for the SPGR sequence. The new TSI sequence achieves the lowest overall relative bias and therefore highest accuracy among the four approaches.

Fig 4 compares the accuracy of *T*_1_ estimates in terms of the relative bias for the four different estimation approaches. We can see that the *T*_1_ estimates using the SPGR methods are biased, with the biases getting more pronounced as SNR decreases. Specifically, at the highest tested SNR level of 480, DESPOT1 has a relative bias of 0.58% and SPGR NLSE of 0.66%. At the lowest tested SNR level of 40, DESPOT1 reaches a relative bias of 53.03% and SPGR NLSE reaches 22.87%. For the same SPGR data, using NLSE to estimate the *T*_1_ value has a lower bias than using the linear data fitting process in DESPOT1. In general, SPGR signals have a relatively high SNR due to short TR. For example, the SNR ranges from 100–200 in brain tissues for the clinical whole-brain SPGR data acquired at 1.5T with a single channel receiver coil (with TR = 8 ms and flip angles of 2°, 3°, 14°, 17°) [11]. As shown in Fig 4, in this SNR region, the SPGR approaches have a relative bias between 4–16%. Note that for a nominal *T*_1_ of 1500 ms, this range of relative bias corresponds to an absolute bias of 60–240 ms. Bias of this range is significant and can severely ambiguate the detection of *T*_1_ changes in NAGM, which was recently shown to be associated with cortical lesions and cognitive dysfunction for patients with long-standing MS [40, 41]. The average *T*_1_ values for normal frontal GM, per the studies in [42], are in the range of 1322ms ± 34 at 3.0T. A *T*_1_ estimate bias on the order of 60–240 ms would imply tissue abnormalities, which actually results from the *T*_1_ relaxometry approach inaccuracies. This finding agrees with the results in [11] that the *T*_1_ values for brain tissues estimated using DESPOT1 can be overestimated by 10–20% in the clinical SPGR images.

In contrast, using the TSI sequences for *T*_1_ relaxometry virtually eliminates the *T*_1_ estimate bias for a broad range of simulated SNRs. At the highest tested SNR level of 60, the original TSI sequence achieves a relative bias of 0.56% and the new TSI sequence of 0.05%. Even at the lowest tested SNR level of 5, the original TSI sequence produces a relative bias of 8.17%, and the new TSI sequence of 4.19%. The clinical SNR of 100–200 for the SPGR sequences translates to an SNR of 12.5–25 after compensating for the SNR equivalence calibration. Fig 4 shows in the SNR range between 12.5–25, the *T*_1_ estimates using the new TSI sequence have a relative bias between 0.25–0.88%. Again, for a nominal *T*_1_ of 1500 ms, this range of relative bias corresponds to an absolute bias of 4–13 ms. Bias of this range may or may not have any clinical significance, given that recent studies showing for NAWM and NAGM, the relevant early *T*_1_ changes are on the order of 10–20 ms for MS patients [30, 31]. However, for clinically realistic SNR levels, the new TSI sequence improves the *T*_1_ estimation accuracy by a factor of 16 times over DESPOT1 for the tested nominal *T*_1_ of 1500 ms. This improvement would greatly alleviate the ambiguities of *T*_1_ variation caused either by the *T*_1_ relaxometry inaccuracies or the underlying pathological conditions of the patients.

### Comparing *T*_1_ sensitivity and orthogonality

As described in the Theory section, the CRB provides a more complete model for the factors controlling *T*_1_ estimate precision. There are three factors contributing to CRB improvement: SNR *M*_0_/*σ*, sensitivity $\left|\right|\frac{\partial h}{\partial T_{1}}\left|\right|$, and orthogonality sin *ϕ*. Improving SNR often requires increasing the *B*_0_ field strength, employing lower noise receiver coils, or increasing the number of signals averaged, all of which increase hardware costs or scan time. Therefore, redesigning pulse sequences to increase the measurement sensitivity $\left|\right|\frac{\partial h}{\partial T_{1}}\left|\right|$ and orthogonality sin *ϕ* can improve the *T*_1_ estimate precision without requiring improved hardware or additional scan time. Here we evaluate and compare the sensitivity and orthogonality terms separately for each pulse sequence and investigate how the different terms contribute to the CRB as *T*_1_ varies.

**Fig 5:**
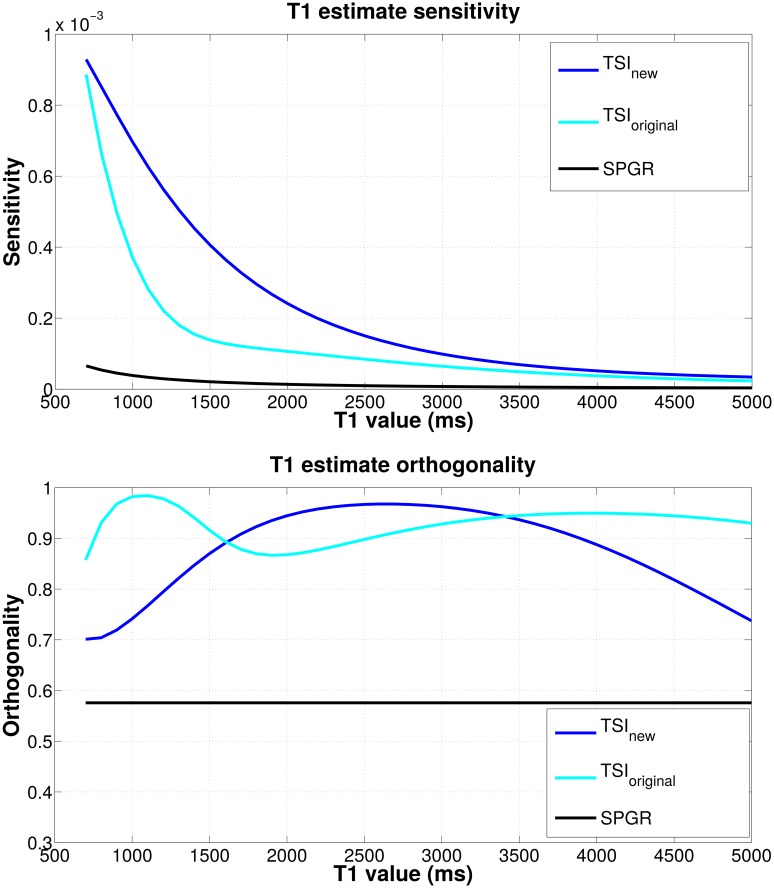
Comparison of sensitivity (top panel) and orthogonality sin *ϕ* (bottom panel) of *T*_1_ estimation for the new TSI sequence (blue), the original TSI sequence (cyan), and the SPGR sequence (black). The norm of *T*_1_ sensitivity $\left|\right|\frac{\partial h}{\partial T_{1}}\left|\right|$ is calculated as the Euclidean norm of the derivative of the signal weighting vector with respect to *T*_1_. Increasing the norm of the sensitivity will increase the impact of small changes in *T*_1_ on the overall signal weighting vector **h**. sin *ϕ* is the orthogonality term defined in Fig 1. The more orthogonal these vectors are, the easier it becomes to ascribe changes in the observed signal to *M*_0_ or *T*_1_ unambiguously. The top panel shows the new TSI sequence has the best sensitivity among the three sequences and SPGR has very poor sensitivity for *T*_1_ estimation. The bottom panel shows the TSI-family sequences have greater orthogonality (above 0.7) than the SPGR sequence (equal to 0.58) for the tested *T*_1_ range.

Fig 5 compares the *T*_1_ sensitivity and orthogonality for the new TSI sequence, the original TSI sequence, and the SPGR sequence as a function of *T*_1_ over the range of 700–5000 ms. The top panel shows the new TSI sequence exhibits the highest sensitivity among the three sequences, especially for the *T*_1_ range of 700–2000 ms corresponding to normal brain tissues. This high sensitivity of the new TSI sequence explains its low mean *T*_1_ estimation error shown in Fig 3. For the original TSI sequence, the sensitivity decreases sharply for *T*_1_ within 700–1500 ms, which implies this sequence is relatively unstable for *T*_1_ relaxometry within the WM and GM regions. For all tested *T*_1_ values, the SPGR sequence has the lowest *T*_1_ sensitivity. The bottom panel in Fig 5 shows the TSI sequences have greater orthogonality than the SPGR sequence. Specifically, within the tested *T*_1_ range, the new TSI sequence has an average orthogonality value of 0.88, compared with the original TSI sequence of 0.92 and the SPGR sequence of 0.57. Comparing the sensitivity, orthogonality, and the equivalent TSI-SPGR SNR factor, we find that the optimized TSI sequence owes its improved *T*_1_ estimation capability to its high *T*_1_ sensitivity and orthogonality of the TSI-family sequences. In contrast, the DESPOT1 sequence owes much of its *T*_1_ estimation capability to its inherently high SNR, largely due to very short TR intervals. The dramatic improved sensitivity and modest improved orthogonality explain the superior performance of the new TSI sequence over DESPOT1 in spite of a conceding factor of 8 in SNR.

## Discussion

### Sequence design approach

Prior to any validation from phantom or in vivo experiments, the Monte Carlo simulation results show that the new TSI sequence achieves improved *T*_1_ precision and accuracy over the popular SPGR approaches. This improvement in precision is prominent for a broad range of brain *T*_1_ values, not only the ones corresponding to normal brain parenchyma, but also to more advanced lesioned tissues and the CSF region. Both *T*_1_ precision and accuracy show desirable stability to *T*_1_ variation and varying SNR levels. The relative mean *T*_1_ estimation errors using NLSE for both the new TSI sequence and the SPGR sequence confirm that the CRB is a reliable approach to predict the performance of a *T*_1_ relaxometry approach.

This work uses a qualitatively new approach in the MRI pulse sequence design, which exploits the Cramér-Rao Bound (CRB) on the variance (stability) achievable by any estimator [28]. This bound-based design allows us to predict the performance of an MRI relaxometry approach based on the pulse sequence parameters, and then optimize the performance of that sequence by adjusting these parameters. We optimized the stability of a *T*_1_ image by finding the pulse times and flip angles that produce the smallest variance for all *T*_1_s within a range of interest. This differs from prior pulse sequence design approaches that either presumed nominal tissue *T*_1_ values *a priori* [14] or focused on improving the signal dynamic range [10, 13] without assessing the implications for the *T*_1_ map stability. Another advantage of the proposed pulse design approach is considering a range of nominal *T*_1_ values in the pulse optimization process. For prior approaches [10, 14], the optimized pulse times and flip angles have strong dependence on the assumed tissue *T*_1_ values. Although *T*_1_ measurements for brain tissues are widely available [32], the inherent *T*_1_ variation within the target tissue degrades the performance of the sequences designed assuming a specific *T*_1_ value for each tissue type. In contrast, the min-max approach provides the best worst case performance, resulting in robust stability for *T*_1_ throughout the region of interest and not just estimates in the neighborhoods of the nominal *T*_1_ values.

From the pulse sequence design perspective, the proposed bound-based framework was derived assuming a general signal model under additive white Gaussian noise. This framework is illustrated with a case study for the TSI sequence to show the effectiveness of the CRB-based framework in optimizing sequence parameters for *T*_1_ relaxometry. However, the proposed framework is readily extendible to other MRI relaxometry sequence designs and sequence parameter optimizations. For example, the proposed framework can be applied to optimize the inversion time and number of data measurements when using the inversion recovery (IR) sequence for *T*_1_ relaxometry. Another example is using the proposed framework to optimize the number of excitation pulses and their inter-pulse times and flip angles when using the Look-Locker (LL) sequence for fast *T*_1_ relaxometry. Alternatively, the proposed framework is also extendible for designing new MRI sequences and optimizing their parameters to simultaneously extract *T*_1_ and *T*_2_ information from the signal measurements. One such example is the DESPOT approach that jointly uses the SPGR and steady state free precession sequences (SSFP) to collect a series of steady state images over a range of flip angles for fast *T*_1_ and *T*_2_ mappings [10]. The proposed framework can be adapted to optimize the flip angles and pulse repetition times for the SPGR and SSFP sequences for optimal precision of joint *T*_1_/*T*_2_ estimation. We believe our pulse design framework is promising for producing sequences with improved stability, precision, and accuracy over a wide range of *T*_1_/*T*_2_ values corresponding to normal tissue and advanced pathologies as well.

### Practical considerations

**Fig 6:**
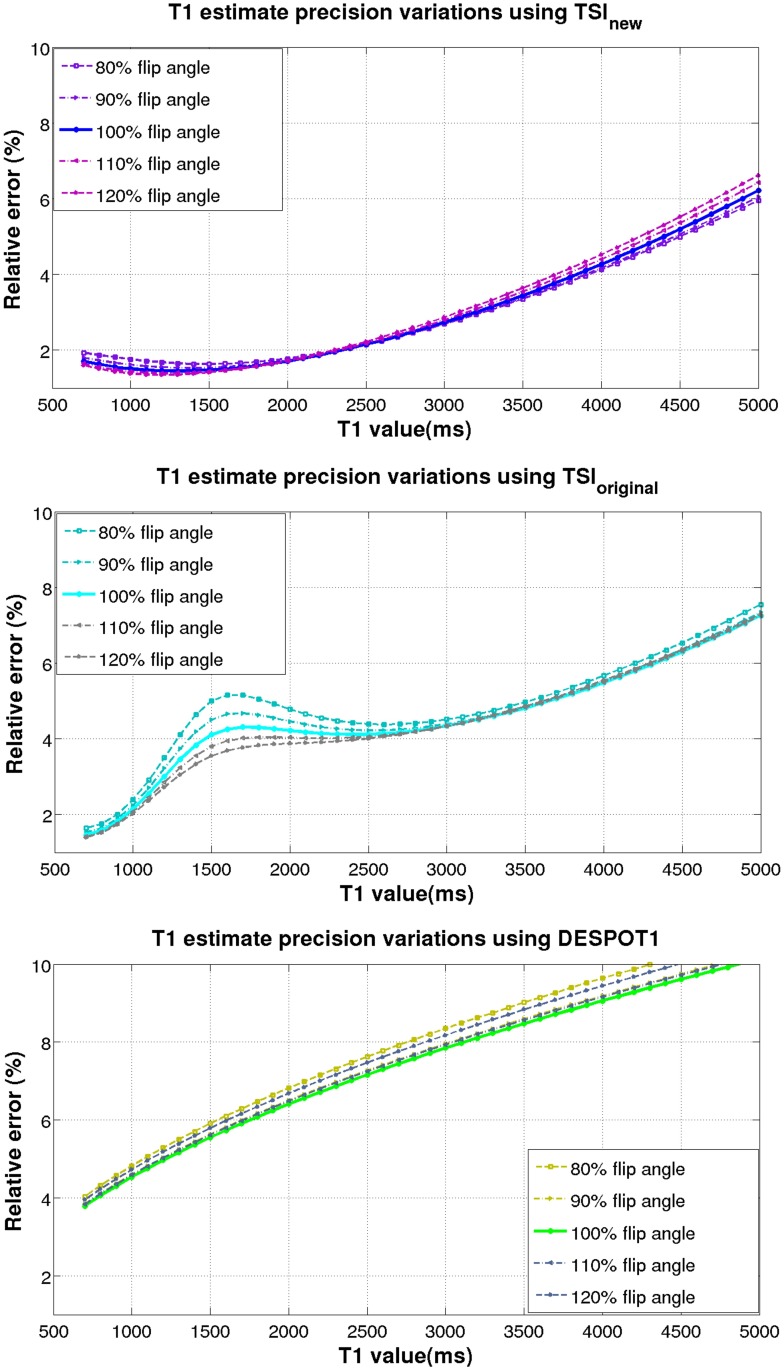
Degradation of the *T*_1_ estimates’ precision in terms of relative errors due to flip angle perturbations using the new TSI sequence at SNR = 125 (top panel), the original TSI sequence at SNR = 125 (middle panel), and the SPGR sequence at SNR = 1000 (bottom panel). The relative error is calculated as the square root of the CRB on *T*_1_ estimates normalized by the true *T*_1_ value. All curves are generated keeping the flip angle of the inversion pulse as 180° and simultaneously varying the flip angles of the imaging pulses within ±20% of their nominal values. Among the three pulse sequences, TSI_new_ is the most robust to flip angle variations for *T*_1_ estimation.

A dominant practical error impacting the *T*_1_ estimation performance of a pulse sequence is the pulse flip angle perturbations. In practice, the flip angle perturbations are mainly caused by patient-induced B1 inhomogeneities due to distortions of the radio-frequency field generated by the transmit coils. Although the use of B1-insensitive adiabatic pulses can result in accurate inversions, the B1 inhomogeneities could affect the imaging pulses with the flip angles perturbations up to ±20% of their nominal values [14]. Observing how the relative error $\sqrt{\text{CRB}}/T_{1}$ degrades due to the flip angle perturbations quantifies how B1 field inhomogeneities affect *T*_1_ estimate precision. Fig 6 demonstrates the degradation of the relative errors due to flip angle perturbations up to ±20% of their nominal values for the new TSI sequence at SNR = 125 (top panel), the original TSI sequence at SNR = 125 (middle panel), and the SPGR sequence at SNR = 1000 (bottom panel). The top panel shows the new TSI sequence is robust to the B1 field inhomogeneities for a wide *T*_1_ range within 700–5000 ms. The flip angle perturbations alter the relative error curve, but not dramatically. Specifically, the relative errors fall within ±0.1% of the unperturbed nominal values for the normal brain tissue range (*T*_1_ 700–2000 ms). For *T*_1_ up to 5000 ms, the relative error still falls within ±0.4% of the unperturbed value of 6.5%. Compared against the new TSI sequence, the flip angle perturbations affect the original TSI sequence more severely. As shown in the middle panel, the relative errors fall within ±0.9% of the unperturbed nominal values for the normal brain tissue range (*T*_1_ 700–2000 ms). The relative error is most sensitive to flip angle perturbations for *T*_1_ of 1300–2100 ms, where most brain GM resides. Compared against the TSI-family sequences, the bottom panel shows that the flip angle perturbations affect the SPGR sequence more severely for all *T*_1_s within 700–5000 ms. This result agrees with the earlier findings on the SPGR-based techniques’ sensitivity to B1 variations [10]. Specifically, for the normal brain tissue range (700–2000 ms), the relative errors fall within ±0.4% of the unperturbed nominal values. As *T*_1_ increases, the relative error degrades more severely (off the chart) and falls within ±0.65% of the unperturbed value of 10.5% for *T*_1_ = 5000 ms. The relative robustness to B1 inhomogeneities and stability to *T*_1_ variation make the new TSI sequence appropriate for studying the effect of diseases such as MS, where the presence of lesions at different degrees of severity may lead to *T*_1_ variations in the order of thousands of milliseconds, rendering measurements by other techniques unreliable.

Regarding the underlying MR physics for different imaging sequences, we assume that the same physical object is scanned by the same MRI scanner under the same physical conditions. The *B*_0_ field strength, the voxel volume size, and the number of data measurements are assumed the same between TSI and DESPOT1. The simulations adjust the distinct SNR levels between TSI and DESPOT1 for different echo times during $T_{2}^{*}$ decay and different receiver bandwidths. However, we ignore several MR physical factors which might affect the performance of the optimized pulse sequence in practice. For example, TSI usually requires about three additional repetitions of the five-pulse sequence before the start of data acquisition to allow magnetization to reach equilibrium condition [14]. Similarly, for the DESPOT1, we have not considered either the time required to reach steady state for each acquisition or the wait time between different sub-sequences in the actual acquisitions. Therefore, a natural next step is to confirm the improved performance of the new TSI sequence in actual MRI experiments. As a first validation, our next effort will focus on phantom experiments with known *T*_1_ values to demonstrate the reliability of the proposed technique.

## Conclusion

This paper designed a new TSI *T*_1_ relaxometry MRI sequence that achieves improved precision and accuracy over a broad range of *T*_1_ values, covering both healthy and lesioned brain tissues. The new sequence demonstrates robustness to B1 inhomogeneity and stability over the wide range of *T*_1_ variations encountered in neuro-degenerative diseases under clinically feasible scan time. This suggests that the improved TSI sequence may be helpful in the study of neuro-degenerative diseases such as multiple sclerosis. The improved performance of the new sequence illustrates the value of the min-max design strategy minimizing the Cramér-Rao bound on *T*_1_ estimation. The geometric interpretation of the Cramér-Rao bound developed here illuminates three factors controlling relaxometry performance: improving the SNR (or signal dynamic range), increasing the signal’s sensitivity to *T*_1_ variation, and increasing the orthogonality between the signal vector and the sensitivity vector. The second and third approaches can be implemented on existing systems to improve the *T*_1_ relaxometry performance without requiring hardware improvements to magnets or measurement coils. The improved relaxometry performance predicted by the Cramér-Rao bound and demonstrated in Monte-Carlo simulations suggests that the proposed benefits of the design strategy may prove portable to other relaxometry sequences.
